# Single-cell RNA sequencing data processing using cloud-based serverless computing

**DOI:** 10.46471/gigabyte.191

**Published:** 2026-09-11

**Authors:** Ling-Hong Hung, Niharika Nasam, Chris Biju, Wes Lloyd, Ka Yee Yeung

**Affiliations:** ^1^ School of Engineering and Technology, University of Washington Tacoma, Tacoma, WA, USA

## Abstract

Single-cell RNA sequencing (scRNA-seq) has become a routine method for measuring cell activities. We present a novel and generalizable methodology using serverless cloud computing to accelerate computationally intensive workflows. We create an on-demand “supercomputer” using rapidly deployable cloud serverless functions as automatically provisioned computation units. We tested our methodology of optimizing an scRNA-seq workflow by leveraging serverless functions on the cloud using two publicly available peripheral blood mononuclear cell (PBMC) datasets. In addition, we demonstrate our approach using a 450 GB human scRNA-seq knockout dataset, comprising 13 samples from different developmental time points, designed to study the temporal impact of perturbations on pancreatic differentiation. We compared the execution time of the scRNA-seq serverless workflow with an optimized workflow without serverless functions running on identical hardware, and demonstrate speedups for all tested datasets, reaching 7.0-fold for the largest dataset. Our software is open source and distributed under the MIT license. Code and documentation are publicly available at  https://github.com/BioDepot/scRNA-serverless.

## Introduction

Single-cell RNA sequencing (scRNA-seq) measures the abundance of mRNA molecules at single cell resolution and has become a routine method in laboratories. The widespread adoption and technological advances of scRNA-seq have led to the development of many computational tools [1]. An scRNA-seq data processing *workflow* typically consists of multiple steps, including data quality control, alignment of sequence fragments to a reference sequence, assignment of reads to genes and cells, cell barcode demultiplexing, UMI deduplication, and counting the number of unique RNA molecules. This procedure results in a gene-by-cell count matrix which is used as an estimate to quantify RNA molecules in each cell for each gene [2]. Data normalization and subsequent downstream analysis are often performed using this gene-by-cell count matrix. Each step in an analytical workflow could have different requirements for computing resources. In particular, many methods and software tools have been developed to perform the computationally intensive alignment step, for example STARsolo [3], Cell Ranger [4], Piscem [5], and Alevin-Fry [6].

Rapid scale-up of scRNA-seq technology (i.e. more cells and samples) coupled with the potential of combining with other data sources at single-cell resolution across modalities (e.g. spatial location) has established the crucial role of scalable computing resources. Cloud computing enables convenient, on-demand access to a shared pool of configurable computing resources that can be provisioned and released as needed. In particular, serverless computing adopts a simplified model to create scalable applications with reduced configuration and management overhead on the cloud [7]. Instead of the user provisioning and managing virtual servers, the cloud provider automatically allocates machine resources as needed [8, 9]. Most public cloud providers offer serverless computing capabilities. The pricing structure for serverless computing is based on actual application runtime, eliminating the cost of renting idle servers. However, serverless functions are designed for small microservices, with limited memory, disk space, and execution time. Here, we present a serverless pipeline for scRNA-seq, where the alignment step is executed through the invocation of multiple serverless functions on the cloud, and demonstrate reduction in execution time.

### Related work

Serverless computing is meant to let businesses and application developers focus on the program they need to run and not worry about the machine(s) it is on or the resources it requires [9]. In serverless computing, server infrastructure is created on demand and dedicated solely to the functions required to execute your code. Serverless computing offers cost savings through a pay-as-you-go model, eliminating the need for provisioning and maintaining dedicated servers. A primary advantage of serverless computing is its propensity for scalability and massive parallel computation. Serverless computing also benefits from the use of flexible cloud services for data storage to provide on-demand access to applications and resources [10].

Grzesik *et al.* [11] reviewed serverless computing solutions in bioinformatics evaluating their usage in omics data analysis and integration. In their survey, they emphasize the utility of serverless computing for providing access to substantial computational resources while supporting the integration of diverse data sources within complex analysis pipelines. Their work highlights the acceleration potential provided by cloud computing, specifically through the serverless paradigm, which streamlines infrastructure management for developers. Their survey offers a critical review of serverless solutions in bioinformatics, while also describing their own application supporting multi-omics data analysis and integration. Notably, their evaluation extends to the effectiveness of these solutions within the context of the COVID-19 pandemic.

Biodepot workflow builder (Bwb) [12] is an open source cloud-enabled containerized platform that simplifies bioinformatics workflow creation through an interactive graphical interface, enabling biomedical researchers to design and customize complex analytical workflows. With drag-and-drop functionality and options to integrate with other open source software such as Jupyter, Fiji and QuPath [13, 14], Bwb streamlines the process of constructing and reproducibly executing complex bioinformatics workflows.

Hung *et al.* [15] introduced an innovative approach achieving a remarkable 1100-fold computational speedup in RNA sequencing analysis. This enabled biomedical scientists to dynamically fine-tune alignment parameters in real-time, facilitating iterative improvements in the final analytical results. They employed a publish and subscribe mechanism to invoke serverless instances to perform the compute intensive alignment step in which millions of short reads (sequences) are mapped to the reference sequence. Efficiency was achieved by splitting the input sequences into smaller pieces (or shards) and invoking more than 1700 serverless instances to perform alignment simultaneously on each data shard, thus resulting in reduced execution time in the alignment step in an RNA-seq workflow. In addition, Hung *et al.* implemented this serverless RNA-seq workflow using the Bwb platform to provide an interactive graphical interface for biomedical scientists [15].

Similar to the approach by Hung *et al.*, Cinaglia *et al.* [10] leveraged serverless computing to support computation of genomic data, focusing on RNA-seq read-mapping to a reference genome, which is the most time-consuming task. Their experiments demonstrated a huge reduction in runtime when the read-mapping was performed on serverless instances, while demonstrating significant enhancements to scalability and parallelism possible with serverless computing.

As another application of serverless techniques in bioinformatics, De Carvalho *et al.* proposed a framework to provision and execute protein sequence alignment workflows composed of multiple tasks [16]. They compared the computation time of workflows executed using traditional virtual machines hosted on AWS EC2 instances with serverless computing using AWS Lambda functions. Their experiments confirmed that protein sequence alignments performed using Lambda functions executed in a shorter average time in relation to the alignment performed on EC2 instances.

Instead of using serverless solutions on the cloud, Chen *et al.* used Nextflow and AWS to build a cloud-native pipeline called Vulture [17]. They demonstrated that Vulture enabled fast, scalable, and cost-effective detection of microbial reads in public scRNA-seq datasets.

While bulk RNA-seq provides estimates of the average expression level for each gene across a population of cells, scRNA-seq can estimate a distribution of expression levels for each gene across a population of cells [18]. A typical scRNA-seq workflow contains the following steps: mapping the short sequences to a reference; assigning reads to genes and cells; and counting the number of unique RNA molecules. The output of these steps includes a gene/cell count matrix, which is often used as input in subsequent downstream analyses (such as visualization, clustering). Examples of clustering methods include Louvain in Seurat [19] and multiscale clustering (MSC) [20]. Another example of downstream analysis, AEnet, combines gene expression levels with alternative splicing patterns to profile cellular heterogeneity [21]. Examples of other multimodal and graph-based biomedical modeling studies include GraCMI [22], CAPTAIN [23], review papers for applications in drug discovery [24], and pangenome graphs [25].

### Our contributions

We present a novel and generalizable methodology using serverless cloud computing to accelerate computationally intensive workflows. Specifically, we present a serverless pipeline for scRNA-seq, where the alignment step is executed through the invocation of multiple serverless function instances. We optimized the Piscem-Alevin Fry pipeline [26] to overcome resource limitations of serverless functions by splitting large input sequence files into smaller chunks and using storage buckets to store input and output files. Each serverless function instance simultaneously maps reads from sequence input files to the reference. The workflow is fully asynchronous: serverless alignment of uploaded shards overlaps the decompression, splitting, and uploading of the remaining input, input objects are streamed directly from storage into the aligner, and aligned outputs are merged using parallel ranged reads. Our results demonstrate speedups over an optimized on-server baseline for all tested datasets, with the largest gains for large scRNA-seq datasets due to the parallel alignment step. We have expanded the serverless approach previously employed by Hung *et al.* [15] in bulk RNA-seq to the realm of scRNA-seq.

## Overview of serverless scRNA-seq workflow

Serverless functions are designed for small short-lived applications and accordingly, have resource limitations. As an example, Amazon Web Services (AWS) Lambda functions are limited to 15 min maximum runtime, and up to 10 GB of memory [27]. In particular, we developed an optimized cloud-based serverless scRNA-seq workflow by leveraging the Piscem-Alevin Fry pipeline [26] that is accurate, scalable, fast, and memory-efficient. Piscem’s process of mapping reads to the reference is considerably faster than conventional aligners, while Alevin-Fry [6] is designed for rapid processing of barcodes and UMIs with minimal memory usage. These features make the pipeline ideal for large-scale scRNA-seq studies, particularly in cloud-based serverless environments where computational and memory efficiency are critical. See the Methods section for a detailed summary of the Piscem-Alevin Fry pipeline [26].

**Figure 1.**
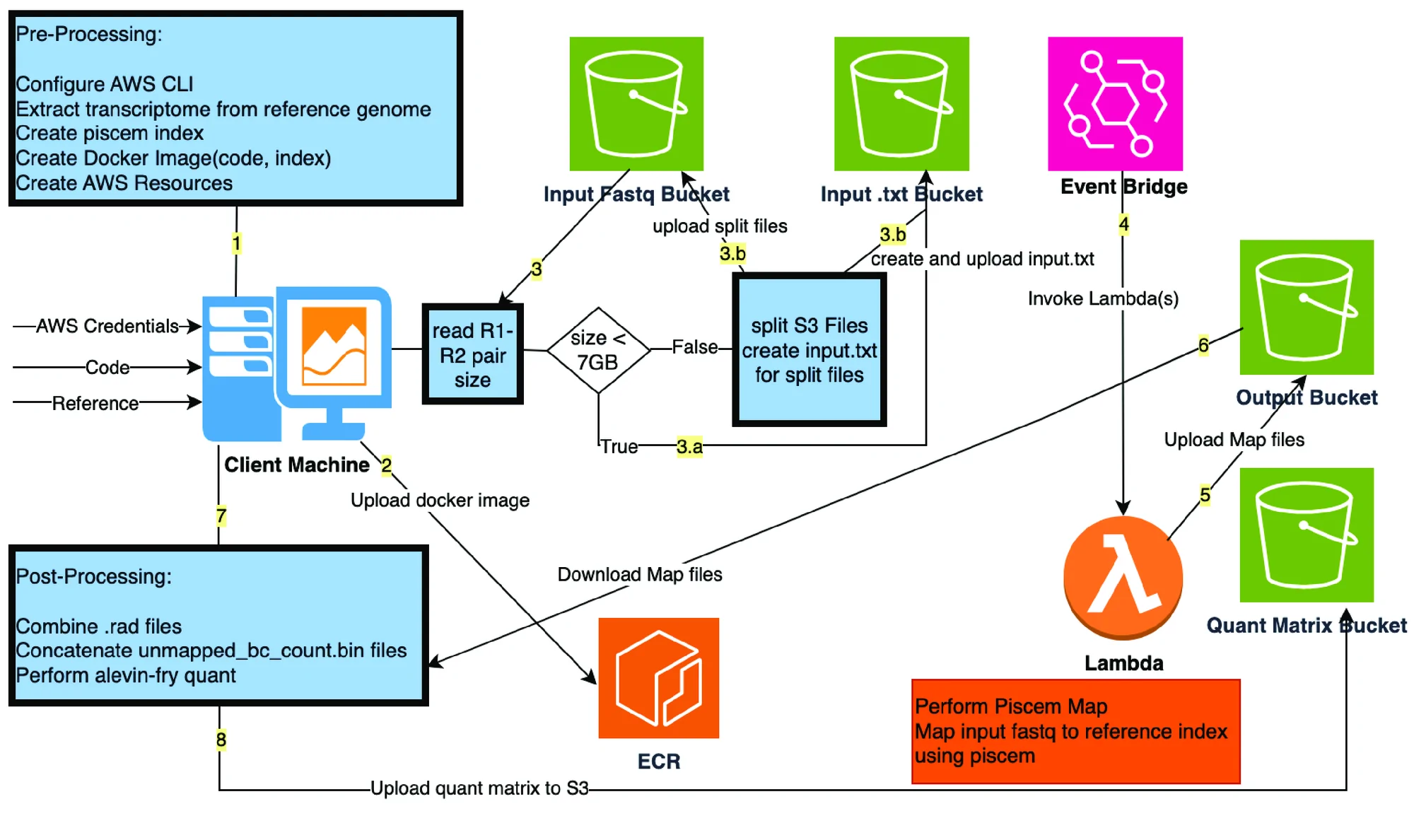
Overview of our optimized Piscem Alevin-Fry serverless architecture. Steps 1, 2 and 3 represent the pre-processing stage where AWS resources are created and input sequence files are decompressed, split into shards, and uploaded when a compressed R1/R2 pair exceeds the 7 GB direct-pass cutoff; smaller pairs are passed to a single Lambda invocation unmodified. Steps 4 and 5 represent serverless execution, which is dispatched asynchronously as each shard is uploaded, with input objects streamed from S3 directly into piscem. Steps 6 and 7 represent the post-processing phase in which shard outputs are merged into the final RAD file(s) using parallel ranged S3 reads before the Alevin-Fry step is run. In step 8, output files from Alevin quant are uploaded to S3. The stages overlap: serverless alignment proceeds while the remaining input is still being split and uploaded.

Our serverless scRNA-seq workflow is implemented using the serverless AWS Lambda function, and the overall architecture is illustrated in Figure 1. The pipeline starts with an initialization step (steps 1, 2, 3 in Figure 1) in which AWS resources are created and input FASTQ files are pre-processed. Due to the small and transient nature of the disk space allotted to a function, a storage bucket (AWS Simple Storage Service S3) is used to store input files that need to be transferred to a function, and output files that are generated by the function that need to be returned to the client. The bucket provides an intermediary for data transfer between the client and the serverless function instances, assuming the role of a distributed file system for a traditional “supercomputer”.

 One of our design challenges is to modify the alignment process so it can execute within the limitations of serverless function instances while simultaneously reducing and mitigating the effect of data transfers between the client, bucket, and serverless layers. This was accomplished by reducing the size of the input FASTQ files if necessary, and by overlapping every stage of the workflow so that no stage waits for the complete output of the previous one. The Lambda function is container-based, with the reference index and executables bundled into the container image. Each function instance is allocated 10 GB of memory and 10 GB of ephemeral storage. Compressed R1/R2 input pairs smaller than a configurable direct-pass cutoff (7 GB) can be passed unmodified to a single Lambda invocation; splitting is reserved for pairs whose work unit would not fit within the local storage of a Lambda instance. Larger pairs are decompressed, split into shards of four million read pairs, and uploaded to S3. Decompression is parallelized at the file level, with each gzip stream assigned one core; only when there are fewer streams than available cores is the multi-threaded decompressor rapidgzip [28] used, so that the spare cores are not left idle. Each shard pair is uploaded as soon as it is materialized, followed by a small manifest file whose upload dispatches an asynchronous Lambda invocation, so that serverless alignment overlaps the remaining decompression, splitting, and uploading. See steps 2 and 3 in Figure 1.

The alignment step in which reads are mapped to the reference is performed using multiple instances of an “alignment” serverless Lambda function executing simultaneously. Rather than staging input files in ephemeral storage before alignment, each Lambda function streams its S3 input objects directly into piscem map [5] through named pipes, so alignment begins before the input transfer completes; streamed byte counts are verified, and truncated transfers are treated as failures. The resulting output files are uploaded to the designated output S3 bucket, followed by a completion marker written only after all outputs are present. Duplicate deliveries of the same shard are suppressed with conditional S3 writes that elect a single owner per output folder. See steps 4 and 5 in Figure 1.

As shard outputs become available, they are merged directly into the final RAD file(s) on the client machine using parallel ranged S3 reads that write each shard payload at its final offset, avoiding a separate download-then-concatenate pass (step 6 in Figure 1). For multi-sample datasets, one RAD file is produced per biological sample, and complete samples are merged eagerly while the remaining samples are still being aligned. The merged output is subsequently processed using Alevin-Fry, which identifies valid cell barcodes, performs barcode correction, and barcode demultiplexing. Finally, alevin-fry quant [29] is executed to perform UMI deduplication and generate the gene expression count matrix (step 7 in Figure 1).

The final quantification matrix is uploaded to an S3 bucket. All AWS resources created during the pipeline setup phase are deleted. Additionally, any split files uploaded to the input FASTQ bucket during processing are removed. See step 8 in Figure 1.

## Data description

We tested our serverless scRNA-seq workflow using three publicly available datasets. Our initial test cases for pipeline validation include two peripheral blood mononuclear cells (PBMCs) datasets provided by 10× Genomics. The first dataset (denoted as **PBMC 1K**) consists of 1,222 PBMCs from a healthy donor, with an approximate size of 5 GB. It is publicly available [30]. The second dataset (denoted as **PBMC 10K**) contains 11,769 PBMCs from a healthy donor, with a larger size of approximately 45 GB. It is publicly available for download [31].

Following the validation phase, a comprehensive analysis is conducted using a dataset generated by the Memorial Sloan Kettering Cancer Center (MSKCC) as part of the NIH MorPhiC (Molecular Phenotypes of Null Alleles in Cells) program [32]. This scRNA-seq dataset (denoted as **KO dataset**) is designed to study the temporal impact of perturbations on pancreatic differentiation. Specifically, it includes knockout (KO) data comprising 13 samples collected at different developmental time points, distributed as 130 R1/R2 FASTQ file pairs. The total dataset size is approximately 450 GB. This dataset is publicly available from the NCBI Gene Expression Omnibus (GEO) with accession number GSE288587 [33] and also at the European Nucleotide Archive (ENA) with accession number PRJEB85005 [34].

The PBMC datasets were used to validate the accuracy of the serverless pipeline and to characterize its performance on smaller inputs, while large-scale benchmarking was performed using the MorPhiC knockout dataset.

## Experimental results

This section presents the execution times of the asynchronous serverless workflow across the **PBMC 1K**, **PBMC 10K**, and **KO** datasets and compares its performance against an optimized on-server execution on identical hardware. The execution time for each processing stage is recorded and analyzed. Each benchmark was repeated three times; all reported values are the arithmetic mean across the three trials, with uncertainties given as the standard error of the mean (SE = sample SD $/\sqrt{3}$ ) in seconds. Per-trial measurements are provided in Supplementary Tables S1 and S2.

### Experimental setup

Both the serverless and the on-server arms were executed on the same on-demand AWS m5dn.8xlarge EC2 instance type (32 vCPUs, 128 GiB RAM, up to 25 Gbps networking) in the us-east-2 region, with the two local NVMe instance-store devices configured as a RAID 0 array with an XFS file system. The instance uses an Intel Xeon Platinum 8259CL processor; AWS does not document the hardware underlying Lambda, but the processor reported inside the Lambda execution environment was the same model, so workflow execution was matched at the processor level as well. Each of the three replicates was executed on a freshly provisioned instance launched from a minimal machine image containing the Piscem index and reference assets; runtime scripts were fetched from a pinned Git revision at boot, and the index was copied to and verified on the NVMe array before any timed measurement. Instance provisioning, dataset transfer, container image construction, and tool installation are excluded from the timed regions. Three on-server baseline trials and three serverless trials were run in separate sessions on fresh instances of the same type and image.

The on-server baseline executes piscem map with 32 threads against the NVMe-resident index and gzipped FASTQ files, timed from invocation to completion. Serverless runs drive the asynchronous workflow from the same instance, uploading to S3 with the AWS CLI configured with default.s3.multipart_chunksize = 256MB (256 MB multipart parts), with each Lambda function instance allocated 10,240 MB of memory and 10,240 MB of ephemeral storage. Lambda runtime measurement starts when the first FASTQ decompressor begins reading the NVMe-resident input and stops when the final RAD file (for the KO dataset, all 13 per-sample RAD files) has been materialized on local storage. The downstream Alevin-Fry steps are excluded from both arms because they are identical in the two workflows.

### Serverless execution time

Table 1 shows the mean execution times for the stages of the asynchronous serverless workflow. During the “split and upload” stage, decompressed shards are uploaded as soon as they are produced and the corresponding Lambda alignment instances are invoked asynchronously, so that serverless alignment runs in parallel with the remaining local work. The “mean Lambda alignment” row reports the mean, over all Lambda invocations, of the interval covering streamed S3 input and piscem map execution. Because this stage runs concurrently with splitting and uploading, its time does not add to that of the other stages. The “post-split wait” stage is the additional time after the final upload until all shard outputs are available in S3, and the “download and merge” stage is the time to materialize the final RAD file(s) locally using parallel ranged S3 reads. The total serverless workflow execution time is the sum of the three driver-side stages: split and upload, post-split wait, and download and merge.

**Table 1:** Mean execution time (in seconds) applying our serverless scRNA-seq pipeline to the PBMC 1K, PBMC 10K, and knockout (KO) datasets. Each entry is the arithmetic mean over three trials in seconds ± the standard error of the mean. The corresponding step number in Figure 1 is shown in column 2. The mean Lambda alignment stage overlaps the split and upload stage and is therefore not additive; the total serverless execution time is the sum of the split and upload, post-split wait, and download and merge stages. The timed region begins when the first FASTQ decompressor starts reading the NVMe-resident input and ends when the final RAD file(s) have been materialized locally; it excludes fetching the raw sequence data to the instance and the Alevin-Fry steps, which are identical in both arms.

Task	Step # in Figure 1	PBMC 1K	PBMC 10K	KO
Files size		4.69 GB	44.05 GB	453.43 GB
Lambda invocations	4	18	161	917
Split and upload [driver]	2, 3	43.7 ± 0.5	377.0 ± 4.5	1146.2 ± 11.4
Mean Lambda alignment [serverless]	4, 5	19.8 ± 0.2	21.0 ± 0.1	31.9 ± 0.2
Post-split wait [driver]	5	18.4 ± 0.6	20.3 ± 0.8	7.7 ± 0.04
Download and merge [driver]	6, 7	1.5 ± 0.1	6.9 ± 0.1	90.9 ± 0.4
**Total serverless execution**		**63.5** **±** **1.2**	**404.3** **±** **4.3**	**1244.8** **±** **11.7**

For the PBMC datasets, whose input pairs are all below the direct-pass cutoff, the pairs were nevertheless decompressed and split into shards of four million read pairs so that alignment is parallelized across multiple invocations, yielding 18 Lambda invocations for PBMC 1K and 161 for PBMC 10K. For the KO dataset, 105 of the 130 R1/R2 pairs fell below the 7 GB direct-pass cutoff and were dispatched unmodified as single invocations, while the remaining 25 pairs were split, for a total of 917 invocations. In a profiled KO trial, all 917 invocations completed with zero errors and zero throttles, 46 cold starts, a peak concurrency of 46 simultaneous instances, and a maximum observed memory footprint of 2,217 MB of the 10,240 MB configured. Because alignment overlaps splitting and uploading, the mean Lambda alignment time of roughly 20 to 32 s contributes almost no additional wall-clock time: the post-split wait is about 20 s for the PBMC datasets and under 8 s for the KO dataset.

### Serverless vs. on-server comparison

Table 2 compares the mean execution time of the serverless pipeline against on-server processing of the PBMC 1K, PBMC 10K, and KO scRNA-seq datasets on the same instance type. The on-server execution runs piscem map with 32 threads against NVMe-resident inputs; the serverless execution time is the total from Table 1. “Speedup” is the ratio of the on-server and serverless arm means; medians and ranges are given in Supplementary Table S2. The serverless pipeline was faster for all three datasets, and the advantage grows with dataset size: 1.27× for PBMC 1K, 1.93× for PBMC 10K, and 7.01× for the KO dataset. For the smallest dataset, the driver-side split and upload stage dominates the serverless execution time, limiting the achievable speedup, whereas for the KO dataset the parallel execution of hundreds of Lambda function instances reduces nearly two and a half hours of on-server alignment to under 21 min.

**Table 2:** Mean execution time (in seconds) comparing our serverless versus on-server processing of the PBMC 1K, PBMC 10K, and knockout (KO) datasets on the same m5dn.8xlarge instance type. Each entry is the arithmetic mean over three trials in seconds ± the standard error of the mean. Speedup is the ratio of the on-server and serverless arm means.

Method	PBMC 1K	PBMC 10K	KO
Files size	4.69 GB	44.05 GB	453.43 GB
Serverless execution	63.5 ± 1.2	404.3 ± 4.3	1244.8 ± 11.7
On-server execution	80.8 ± 0.3	780.5 ± 0.7	8727.7 ± 15.8
**Speedup**	**1.27×**	**1.93×**	**7.01×**

### Execution cost

Table 3 reports the estimated cost of both arms as the mean over the three trials. The on-server cost is the EC2 instance time of the baseline; the serverless cost is the EC2 instance time of the driver plus the Lambda and S3 charges. EC2 time is charged at the on-demand m5dn.8xlarge rate in us-east-2 ($2.176 per hour), prorated by the measured wall-clock seconds. Lambda charges apply the ×86 list prices for compute, requests, and ephemeral storage to the billed durations recorded by CloudWatch at the configured 10,240 MB of memory and ephemeral storage. S3 write requests ($0.005 per 1,000 PUT and POST requests) are counted exactly from the multipart part counts recorded in the ETags of the retained objects and from the claim messages in the CloudWatch logs. The workflow uploads 23.8 GB, 228 GB, and 1,476 GB of input objects for PBMC 1K, PBMC 10K, and KO, respectively (decompressed shards, plus 257 GB of directly passed gzip pairs for KO), and 1.3 GB, 11.6 GB, and 114 GB of shard outputs. Data transfer within the region is free, and the remaining S3 charges (GET, HEAD, and once-per-second listing requests, and storage for the duration of the run, assuming the objects are deleted on completion) are bounded below $0.06 for KO and $0.01 for the PBMC datasets; they are excluded, as are other ancillary services, free-tier allowances, Savings Plans, and credits.

**Table 3:** List-price cost estimates in USD (us-east-2), mean over three trials, for the on-server and serverless arms. EC2 costs prorate the on-demand hourly rate by the measured wall-clock time; Lambda costs are computed from CloudWatch billed durations at 10,240 MB memory and 10,240 MB ephemeral storage; S3 costs count the write requests recorded in the multipart ETags of the retained objects and in the claim log messages; uploads from the driver used the AWS CLI setting default.s3.multipart_chunksize = 256MB (aws configure set default.s3.multipart_chunksize 256MB) with the default 8 MB multipart threshold. S3 read, listing, and short-lived storage charges (bounded below $0.06), other ancillary services, free-tier allowances, credits, and taxes are excluded.

	PBMC 1K	PBMC 10K	KO
On-server EC2	$0.049	$0.472	$5.275
Serverless driver EC2	$0.038	$0.244	$0.752
Serverless Lambda	$0.064	$0.585	$5.078
Serverless S3	$0.002	$0.022	$0.156
**Serverless total**	$**0.105**	$**0.851**	$**5.986**
Cost ratio	2.14×	1.80×	1.13×

Although the serverless workflow is faster for every dataset, it is more expensive at list prices because the aggregate Lambda compute exceeds the driver EC2 time saved. The cost premium shrinks as the dataset grows: 2.14× for PBMC 1K, 1.80× for PBMC 10K, and 1.13× for KO. For the largest dataset, the serverless workflow therefore delivers a 7.0-fold reduction in turnaround time for about 13% more cost. An unused monthly Lambda free tier (one million requests and 400,000 GB-s per month) would substantially reduce the Lambda charges, but the estimates do not assume that this account-level allowance is available.

### Output validation

The serverless and on-server arms produced identical results for every dataset. The total and mapped read counts were identical in both arms: 66,601,887 reads (40,832,376 mapped) for PBMC 1K; 638,901,019 reads (369,342,136 mapped) for PBMC 10K; and 6,623,775,561 reads (3,645,770,776 mapped) for the KO dataset, with equality additionally verified for each of the 13 KO samples. Every merged RAD file passed structural validation, the sorted alignment records were identical between the arms, and the final count matrices were identical after normalizing barcode order (see the Methods section).

## Discussion

We have demonstrated how serverless cloud instances can be harnessed to provide on-demand and scalable “supercomputing” without the need for specialized hardware or a large cluster of server nodes. Our experimental results show that the asynchronous serverless workflow outperforms an optimized 32-thread on-server execution on identical hardware for all three datasets, with the advantage growing with dataset size: 1.27× for PBMC 1K, 1.93× for PBMC 10K, and 7.01× for the 450 GB KO dataset. Overlapping the workflow stages is central to this result: because Lambda instances are invoked asynchronously as each shard is uploaded, inputs are streamed directly from S3 into the aligner, and completed outputs are merged eagerly, the serverless alignment step adds only seconds of wall-clock time beyond the driver-side splitting and uploading. For small datasets, the split and upload stage dominates, so the speedup is modest; the on-demand parallelism pays off most for large inputs, where hundreds of concurrent function instances replace hours of on-server alignment.

The size of the work unit assigned to each Lambda invocation depends on the dataset. The compressed input pairs of the smaller PBMC datasets are below the direct-pass cutoff and would otherwise each be processed by a single invocation, so they are split into shards of four million read pairs to obtain a useful degree of parallelism. The shard size is chosen so that each invocation performs at least about 20 s of alignment, which keeps the fixed startup overhead of an invocation small relative to its useful work. Reducing the shard size further would increase parallelism but with diminishing returns, because each additional invocation incurs cold-start latency, per-shard S3 transfer overhead, and an additional output file to be merged on the driver. For large datasets such as KO, which consist of many input file pairs, it is more efficient to maximize the work done on Lambda: whole compressed pairs are passed directly to the function, so that decompression is performed by the Lambda instances and is therefore fully parallelized rather than performed on the driver. Splitting is reserved for pairs whose work unit would not fit within the local storage of a Lambda instance. When splitting is required and there are more available cores than gzip streams, the driver decompresses each stream with rapidgzip, a multi-threaded decompressor, to utilize the extra cores.

The serverless arm costs more than the on-server baseline (2.14× for PBMC 1K, 1.80× for PBMC 10K, and 1.13× for KO) because the aggregate Lambda compute exceeds the driver EC2 time that is saved. The premium shrinks with scale, however, and for the KO dataset a 7.0-fold reduction in turnaround time costs about 13% more. Whether this trade-off is favorable depends on the value of reduced turnaround time and on account-level discounts such as the Lambda free tier, Savings Plans, or credits.

The multipart part size used for S3 uploads has a measurable effect on both cost and time, because S3 bills every request rather than every byte transferred. With the AWS CLI default of 8 MB parts, the same workflow measured 61.2 ± 0.6 s, 369.5 ± 2.0 s, and 1,314.6 ± 3.9 s for PBMC 1K, PBMC 10K, and KO (Supplementary Table S3), but its KO input uploads alone required about 181,000 S3 write requests ($1.02). Raising the part size to 256 MB removed 95% of those requests, saving $0.86 per KO run, and shortened the KO run by 5% because its many concurrent file and shard uploads retain their natural parallelism. The PBMC datasets, whose two lanes yield only four input streams, slowed by 4% and 9% because fewer, larger parts reduce the upload concurrency available to each stream; an intermediate part size of 64 MB is expected to recover this loss while still reducing the request count eight-fold relative to the default.

These findings suggest that serverless workflows are particularly attractive for processing large-scale genomic data, where they deliver large reductions in turnaround time at a modest cost premium, while for small datasets the gains are limited by driver-side preprocessing. A systematic investigation to determine impacts of various configuration parameters (shard size, direct-pass cutoff, Lambda concurrency, Lambda memory, EC2 instance type, Piscem threads, data transfer, output-merging overhead, etc.) for different datasets represents future work.

## Methods

### Steps involved in single-cell RNA sequencing

Several computational steps are generally involved in processing raw scRNA-seq data into a gene-by-cell count matrix. In the *transcriptome annotation step*, the reference is annotated using a gene annotation file, which provides the locations of genes and transcripts. This step is crucial for accurately mapping sequencing reads to known genes. The *transcriptome indexing* step facilitates rapid and efficient read alignment by creating an index for the annotated transcriptome. Indexing allows alignment tools to quickly search and match sequencing reads to reference sequences. In the *read alignment step*, sequenced reads are aligned to the indexed reference transcriptome. This step assigns each read to its most likely transcript, enabling gene-level quantification. Next, barcodes are filtered based on the cumulative frequency distribution of reads associated with each barcode. This *permit list generation* step ensures that only valid barcodes representing real cells are retained. In the *barcode correction* step, sequencing errors in cell barcodes are corrected by matching them to the closest accepted barcodes. This improves data accuracy by reducing barcode mismatches. Next, reads are grouped according to their corrected cell barcodes, assigning each read to its respective single cell. This *barcode demultiplexing* step enables downstream cell-specific analysis. Unique Molecular Identifiers (UMIs) are used to remove duplicate reads that share the same barcode, UMI, and gene combination. This *UMI deduplication* step prevents amplification bias from affecting gene quantification. Finally, a gene expression count matrix is generated, where rows correspond to genes, columns correspond to cells, and values represent the number of times a gene is detected in a given cell. This matrix serves as the foundation for downstream analyses such as clustering and differential expression analysis.

### Piscem-Alevin Fry pipeline

The Piscem-Alevin Fry pipeline [26] is a highly efficient and scalable solution for processing scRNA-seq data. Alevin-Fry is not only faster and more memory-efficient than other accurate quantification methods, it also improves memory scalability and reduces false-positive expression issues found in other lightweight tools [6]. Piscem is a lightweight mapping tool optimized for speed and memory efficiency, making it well suited for cloud-based serverless execution. Alevin-Fry [6], a companion tool, is designed to efficiently handle barcode correction, UMI deduplication, and count matrix generation, further enhancing the overall performance of the workflow [26].

The pipeline takes as input raw sequencing data in the form of FASTQ files, along with a reference genome file. The workflow begins with transcriptome annotation, where genes are mapped onto the reference genome to establish a structured representation of gene locations. Next, a transcript-to-gene mapping file is generated, providing the necessary reference for downstream analysis.

The first computational step in the pipeline involves indexing the transcriptome using the command piscem build. This indexing step is crucial for accelerating the alignment process in subsequent steps. Once the index is prepared, raw sequencing reads are mapped to the reference transcriptome using piscem map, a highly optimized mapping algorithm that efficiently aligns reads while maintaining accuracy.

Following the mapping step, Alevin-Fry is used to process and refine the data. The first step, executed via alevin-fry generate-permit-list [35], involves generating a permit list, which identifies valid cell barcodes from the sequencing data. This is followed by the alevin-fry collate [36] step, where barcode correction is performed to adjust for sequencing errors, and barcode demultiplexing is applied to assign reads to individual cells.

The final step in the pipeline is gene quantification. The alevin-fry quant [29] command performs UMI deduplication, ensuring that duplicate reads originating from PCR amplification are removed. This step also generates the final count matrix, where each row represents a gene, each column represents a single cell, and each value corresponds to the number of times a gene was detected in that specific cell.

### Implementation: serverless scRNA-seq workflow on the cloud

The pipeline begins with the input parameters, which include Amazon Web Services (AWS) credentials, code, and a reference genome file. The input FASTQ files are stored in an S3 [37] bucket, labeled as **Input FASTQ Bucket** in Figure 1. The first step in the workflow involves configuring the AWS Command Line Interface (CLI) on the client machine. Once configured, the transcriptome is annotated from the reference genome, followed by the generation of a transcript-to-gene mapping file. The annotated transcriptome is then indexed using piscem index. The transcriptome index is bundled with the pipeline’s code, and a Docker image is created; on the cloud, AWS access is granted through an IAM instance profile rather than credentials baked into the image.

#### Creation of cloud resources

The next step involves creating the necessary AWS resources. An Amazon Elastic Container Registry (ECR) [38] repository is created to store the Docker image. The image is built locally and then pushed to the ECR repository. This is labeled as step 1 in Figure 1. A Lambda [27] execution role is defined with the required policies to grant access to S3 resources. Subsequently, a Lambda function is created and configured to execute containerized workloads. Several S3 buckets are also set up: the **output bucket** to store Lambda-generated outputs, the **input .txt files bucket** to hold generated input.txt manifest files, and the **final output bucket** to store the final Alevin-Fry quantification results. When the event-triggered dispatch path is used, an EventBridge rule is also created to invoke the Lambda function upon manifest uploads to the input .txt bucket, and appropriate permissions are granted.

Once AWS resources are configured, the client machine examines each R1/R2 file pair. Compressed pairs smaller than the configurable direct-pass cutoff (7 GB) are dispatched unmodified: an input.txt manifest containing the S3 paths of the pair is uploaded, and the corresponding Lambda invocation streams and decompresses the gzip inputs directly. Larger pairs are decompressed and split into shards of four million read pairs (16 million FASTQ lines per mate) before upload. Decompression is parallelized: when there are fewer gzip streams than available CPUs, each stream is decompressed with rapidgzip [28] using up to eight threads; otherwise, each stream receives a single core and uses gzip. Larger pairs are admitted first, and the R1 and R2 streams of a pair release their cores independently as they finish. Each completed shard pair is uploaded immediately with the AWS CLI, using 256 MB multipart parts, followed by its input.txt manifest, so that Lambda alignment overlaps the remaining local decompression, splitting, and uploading. To maintain uniqueness, each input.txt filename includes extracted lane and part information. These are labeled as steps 2 and 3 in Figure 1.

#### Lambda configuration and execution

The Lambda function in this pipeline is container-based and is configured to pull the image stored in the ECR repository. The function is allocated 10,240 MB of memory and 10,240 MB of ephemeral storage. Lambda executions are dispatched asynchronously as each manifest is uploaded, either through an EventBridge [39] rule that fires on manifest uploads to the input .txt bucket or by direct asynchronous invocation from the client; in both cases, S3 remains the data plane and the client does not wait for individual invocations to complete.

Upon execution, the Lambda function reads the input.txt manifest and creates one named pipe (FIFO) per R1/R2 input. Concurrent S3 GetObject readers write the object bodies into the FIFOs while piscem map [5] consumes them, so alignment begins before the inputs have finished transferring and the inputs are never staged in ephemeral storage. Both uncompressed FASTQ shards and directly passed .fastq.gz inputs are supported; for the latter, Piscem performs the gzip decoding. Streamed byte counts are verified against the S3 object sizes, and truncated transfers are treated as failures. The resulting output files are uploaded to the designated output S3 bucket, and an output.txt marker written after all shard outputs serves as the durable completion signal. To ensure unique output directory names, each output folder is named using the extracted lane and part information from the input file pairs. These are labeled as steps 4 and 5 in Figure 1.

Because asynchronous events can be delivered more than once, duplicate processing is suppressed with conditional S3 writes: a Lambda instance claims an output folder by writing a claim object with If-None-Match, renews a 180-s lease every 30 s using the claim ETag while it works, and conditionally releases only the claim it owns on failure; an expired lease can be taken over by a later delivery. The completion marker, not the claim, is the readiness contract used by the client. This mechanism provides idempotency under at-least-once event delivery rather than general distributed transaction semantics.

#### Post-processing and quantification

As the client machine polls S3, a shard is considered ready when both its map.rad output and its output.txt completion marker exist. Ready shard outputs are merged into the final RAD file by s3-rad-materialize, a C++ AWS SDK client that inspects the shard RAD preludes, constructs a single valid final prelude, and uses parallel ranged S3 reads to write each shard payload directly at its final offset in the output file, avoiding a separate download-then-concatenate pass. The per-shard unmapped_bc_count.bin files are concatenated into a single file. For multi-sample datasets such as KO, an explicit sample manifest maps each input pair to its biological sample and one RAD file is produced per sample; up to four complete samples are materialized concurrently with eight transfer threads each, with readiness tracked through a single global S3 inventory rather than per-sample bucket listings. This is labeled as step 6 in Figure 1.

The merged files are then processed using Alevin-Fry. The first step involves running alevin-fry generate-permit-list [35], which identifies valid cell barcodes based on the cumulative frequency distribution of reads associated with each barcode. Next, alevin-fry collate [36] is executed to perform barcode correction and barcode demultiplexing. Finally, alevin-fry quant [29] is executed to perform UMI deduplication and generate the gene expression count matrix. This is labeled as step 7 in Figure 1.

The final quantification matrix is uploaded to the Quant Matrix S3 bucket to store final results. After this step, all AWS resources created during the pipeline setup phase are deleted. Additionally, any split files uploaded to the input FASTQ bucket during processing are removed, restoring the input bucket to its original state before pipeline execution. This is labeled as step 8 in Figure 1.

#### Validation and sanity checks

The serverless and on-server arms were compared at three levels. First, the total and mapped read counts reported by Piscem must be identical in both arms for every dataset and, for the KO dataset, for every individual sample. Second, every merged RAD file is structurally validated with radtk view. Because Piscem worker scheduling may alter record order, chunk boundaries, and the number of chunk headers between runs, RAD-level comparisons canonicalize the records by sorting before comparison; the sorted record multisets were identical between the arms. Third, the final Alevin-Fry count matrices, cell barcode lists, and gene lists are compared after normalizing barcode order, because parallel shard completion can change barcode ordering without changing counts. As an additional determinism check, re-merging retained KO shard outputs with an unchanged ordered shard manifest produced byte-identical per-sample RAD files.

To support reproduction, we provide a fully automated end-to-end orchestration script that provisions all required cloud resources, executes the serverless pipeline, downloads results, and cleans up infrastructure without manual intervention; the script automatically detects account-specific AWS service quotas (such as the maximum Lambda memory and concurrency and the available EC2 instance types) and adapts the shard size, thread count, and instance selection accordingly. The released script and a step-by-step reproduction guide are available in the project repository.

## Availability of source code and requirements

- Project name: Serverless single-cell RNA sequencing workflow
- Project home page: https://github.com/BioDepot/scRNA-serverless
- Operating system(s): Ubuntu
- Programming language: Shell script, Python
- Other requirements: Docker
- License: MIT
- Cloud IDE: A pre-configured GitHub Codespace is provided for running the on-server (non-serverless) pipeline without any local software installation. The repository includes a .devcontainer configuration that automatically provisions the required environment. When creating a Codespace, select the default 2-core machine and US West region. A Codespace can be launched directly from the repository at https://codespaces.new/BioDepot/scRNA-serverless.
- Piscem reference index for our pipeline is available at Zenodo with https://doi.org/10.5281/zenodo.19375096 [40].

## Data Availability

The knockout (KO) data supporting the results of this article are available in the NCBI Gene Expression Omnibus (GEO) repository with accession number GSE288587 [33] and also at the European Nucleotide Archive (ENA) with accession number PRJEB85005 [34]. The **PBMC 1K** data are publicly available [30]. The **PBMC 10K** data are publicly available [31]. The gene-by-cell count matrices produced by the serverless and by the conventional workflows for the PBMC 1K and PBMC 10K datasets are deposited at Zenodo [41].

## List of abbreviations

AWS, Amazon Web Services; ECR, Elastic Container Registry; IAM, Identity and Access Management; KO, knockout; MorPhiC, Molecular Phenotypes of Null Alleles in Cells; PBMC, peripheral blood mononuclear cell; RAD, reduced alignment data; SE, standard error; UMI, unique molecular identifier.
