# Supplementary material for: Single-cell RNA sequencing data processing using cloud-based serverless computing
Source: GigaByte. 2026 Sep 11;2026:gigabyte191. doi: 10.46471/gigabyte.191 (PMC13613206; doi:10.46471/gigabyte.191)
Supplement: Peer review history

# Reviewer report: Review MS


Reviewer name and names of any other individual's who aided in reviewerBoya JiDo you understand and agree to our policy of having open and named reviews, and having your review included with the published manuscript. (If no, please inform the editor that you cannot review this manuscript.)YesIs the language of sufficient quality?YesPlease add additional comments on language quality to clarify if neededIs there a clear statement of need explaining what problems the software is designed to solve and who the target audience is? YesAdditional CommentsIs the source code available, and has an appropriate Open Source Initiative license <a href="https://opensource.org/licenses" target="_blank">(https://opensource.org/licenses)</a> been assigned to the code?NoAdditional CommentsAs Open Source Software are there guidelines on how to contribute, report issues or seek support on the code?NoAdditional CommentsIs the code executable?Unable to testAdditional CommentsIs installation/deployment sufficiently outlined in the paper and documentation, and does it proceed as outlined?Unable to testAdditional CommentsIs the documentation provided clear and user friendly?YesAdditional CommentsAdditional CommentsIs there a clearly-stated list of dependencies, and is the core functionality of the software documented to a satisfactory level?YesAdditional CommentsHave any claims of performance been sufficiently tested and compared to other commonly-used packages? Not applicableAdditional CommentsAdditional CommentsAre there (ideally real world) examples demonstrating use of the software? NoAdditional CommentsAdditional CommentsAny Additional Overall Comments to the AuthorThe manuscript addresses an important and practical problem in scalable scRNA-seq data processing. I recommend acceptance after revision： 1，The current comparison uses different hardware settings for the serverless and on-server workflows, which weakens the fairness of the speedup claim. The authors should add a controlled benchmark using the same input, same Piscem-Alevin Fry parameters, same storage type, and clearly matched CPU/thread resources. 2，Since the main advantage of serverless computing is elastic, pay-as-you-go execution, the manuscript should include a cost breakdown for EC2, Lambda, S3 storage, data transfer, and ECR usage. A “cost per GB” or “cost per million reads” metric would make the practical value much clearer. 3，The paper states that count matrices are identical in constrained runs, but it should also compare serverless and non-serverless outputs using cell barcode overlap, gene count correlation, UMI totals, detected genes per cell, and downstream clustering consistency. This would show that acceleration does not alter biological results. 4，The manuscript would benefit from a sensitivity analysis of shard size, Lambda memory, thread number, concurrency limit, S3 transfer time, and merge overhead. This would help readers understand when serverless processing is beneficial and when I/O or orchestration overhead becomes the bottleneck. 5，The Related Work section should cite recent computational biology and multimodal/graph-based biomedical modeling studies to better position the work within modern bioinformatics computing: 10.1016/j.patcog.2025.112078； 10.1038/s41467-026-72882-y； 10.1093/nsr/nwaf495； 10.1186/s13059-025-03606-6RecommendationMajor Revisions


# Reviewer report: Review MS


Reviewer name and names of any other individual's who aided in reviewerYang ZhangDo you understand and agree to our policy of having open and named reviews, and having your review included with the published manuscript. (If no, please inform the editor that you cannot review this manuscript.)YesIs the language of sufficient quality?YesPlease add additional comments on language quality to clarify if neededIs there a clear statement of need explaining what problems the software is designed to solve and who the target audience is? YesAdditional CommentsIs the source code available, and has an appropriate Open Source Initiative license <a href="https://opensource.org/licenses" target="_blank">(https://opensource.org/licenses)</a> been assigned to the code?YesAdditional CommentsAs Open Source Software are there guidelines on how to contribute, report issues or seek support on the code?YesAdditional CommentsIs the code executable?YesAdditional CommentsIs installation/deployment sufficiently outlined in the paper and documentation, and does it proceed as outlined?YesAdditional CommentsIs the documentation provided clear and user friendly?YesAdditional CommentsIs there enough clear information in the documentation to install, run and test this tool, including information on where to seek help if required?YesAdditional CommentsIs there a clearly-stated list of dependencies, and is the core functionality of the software documented to a satisfactory level?YesAdditional CommentsHave any claims of performance been sufficiently tested and compared to other commonly-used packages? YesAdditional CommentsIs test data available, either included with the submission or openly available via cited third party sources (e.g. accession numbers, data DOIs)?YesAdditional CommentsAre there (ideally real world) examples demonstrating use of the software? YesAdditional CommentsIs automated testing used or are there manual steps described so that the functionality of the software can be verified?YesAdditional CommentsAny Additional Overall Comments to the AuthorThis study developed an AWS Lambda–based serverless workflow for single-cell RNA sequencing data processing. The computationally intensive read-mapping step of the Piscem–Alevin-Fry pipeline was distributed across multiple Lambda instances, while Amazon S3 was used for data transfer and result aggregation. The workflow was evaluated using two PBMC datasets and a MorPhiC knockout dataset of approximately 453 GB. The results showed that the serverless workflow achieved up to a 6.45-fold speedup for large datasets, whereas it was slower than conventional server-based processing for small datasets because of cloud resource initialization and data transfer overhead. The authors also implemented automatic AWS quota detection and resource fallback mechanisms and demonstrated that identical expression matrices could still be generated under constrained account settings. Overall, the work has practical engineering value, although the fairness of the performance comparison, cost-effectiveness, and validation of output accuracy require further improvement. Specific Comments 1. The serverless workflow was orchestrated using an AWS m6id.16xlarge instance with 64 vCPUs, 256 GB RAM, and high-speed NVMe storage, whereas the conventional workflow was run on a local server equipped with an Intel i9-13900KF processor and 125 GB RAM. The two environments differ substantially in CPU architecture, core count, storage, networking, and overall computational capacity. Therefore, the reported speedup cannot be attributed solely to the serverless parallelization strategy. The authors are encouraged to add a comparison under the same cloud environment and a comparable computational resource budget, or at least discuss and normalize the impact of these hardware differences. 2. The manuscript repeatedly emphasizes the pay-as-you-go model and potential cost advantages of serverless computing, but only execution time is reported. No actual costs are provided for AWS Lambda, EC2, S3 storage, data transfer, or ECR. For practical users, financial cost is as important as runtime. The authors should report the total cost of processing each dataset and compare it with the cost of conventional EC2-based or dedicated-server processing. 3. The authors showed that identical count matrices were generated under different AWS quota settings, which mainly demonstrates reproducibility of the same workflow under different resource configurations. A more rigorous comparison with the standard, non-sharded Piscem–Alevin-Fry workflow is needed. The authors should compare the resulting gene expression matrices, detected cell numbers, total UMI counts, numbers of detected genes, and preferably downstream clustering results to demonstrate that FASTQ splitting and RAD file merging do not introduce computational bias. 4. The reported runtimes appear to be based on single executions, without means, standard deviations, or confidence intervals. Serverless performance can vary because of Lambda cold starts, concurrent scheduling, network transfer, and fluctuations in cloud platform load. Each experiment should be repeated multiple times, and the authors should report runtime distributions, variability, and, where possible, the contribution of cold-start latency. 5. The PBMC 1K dataset of 4.69 GB was processed more slowly using the serverless workflow, whereas the 44.05 GB and 453.43 GB datasets showed clear speedups. The authors should evaluate additional datasets or controlled subsets of different sizes to estimate the point at which serverless processing becomes more efficient than conventional computing. The effects of shard size, Lambda concurrency, data transfer time, and output-merging overhead should also be systematically analyzed. In addition, the phrase “Singe cell” in the title and throughout the manuscript should be corrected to “Single-cell.”RecommendationMinor Revisions


# Reviewer report: Re-Review MS


Indicate in the comments box below whether you are happy with the changes made or if the manuscript is unacceptable.Comments on revised manuscriptThe authors have addressed my concerns, and I recommend acceptance for publication.Indicate in the comments box below whether you are happy with the changes made or if the manuscript is unacceptable.Comments on revised manuscriptThe authors have addressed my concerns, and I recommend acceptance for publication.


# Editor report: Accept


Editor’s AssessmentThe manuscript is ready for formal acceptance.Editor’s AssessmentThe manuscript is ready for formal acceptance.
